# Treatment of Highly Pathogenic H7N9 Virus-Infected Mice with Baloxavir Marboxil

**DOI:** 10.3390/v11111066

**Published:** 2019-11-15

**Authors:** Maki Kiso, Seiya Yamayoshi, Yuri Furusawa, Masaki Imai, Yoshihiro Kawaoka

**Affiliations:** 1 Division of Virology, Department of Microbiology and Immunology, Institute of Medical Science, University of Tokyo, Minato-ku, Tokyo 108-8639, Japan; 2 Department of Pathobiological Sciences, School of Veterinary Medicine, University of Wisconsin–Madison, Madison, WI 53706, USA; 3 Department of Special Pathogens, International Research Center for Infectious Diseases, Institute of Medical Science, University of Tokyo, Minato-ku, Tokyo 108-8639, Japan

**Keywords:** influenza, baloxavir marboxil, H7N9, highly pathogenic

## Abstract

Viral neuraminidase inhibitors show limited efficacy in mice infected with H7N9 influenza A viruses isolated from humans. Although baloxavir marboxil protected mice from lethal challenge infection with a low pathogenic avian influenza H7N9 virus isolated from a human, its efficacy in mice infected with a recent highly pathogenic version of H7N9 human isolates is unknown. Here, we examined the efficacy of baloxavir marboxil in mice infected with a highly pathogenic human H7N9 virus, A/Guangdong/17SF003/2016. Treatment of infected mice with a single 1.5 mg/kg dose of baloxavir marboxil protected mice from the highly pathogenic human H7N9 virus infection as effectively as oseltamivir treatment at 50 mg/kg twice a day for five days. Daily treatment for five days at 15 or 50 mg/kg of baloxavir marboxil showed superior therapeutic efficacy, largely preventing virus replication in respiratory organs. These results indicate that baloxavir marboxil is a valuable candidate treatment for human patients suffering from highly pathogenic H7N9 virus infection.

## 1. Introduction

Treatment of zoonotic influenza with neuraminidase (NA) inhibitors that target the viral sialidase activity of NA is thought to be effective. However, the efficacy of these inhibitors against H7N9 viruses is limited in mice [1,2]. In 2018, a novel antiviral, baloxavir marboxil, which targets the viral endonuclease activity of PA, was approved in several countries for the treatment of seasonal influenza. Baloxavir marboxil is also considered to be an antiviral drug against emerging zoonotic influenza viruses. In patients infected with a seasonal influenza virus, baloxavir treatment reduced the viral load after only 1 day of treatment, which is more rapid than treatment with an NA inhibitor; however, the time to symptom improvement was similar between baloxavir marboxil and NA inhibitor treatments [3]. After baloxavir marboxil treatment, mutant viruses harboring the PA-I38T/M/F mutation with reduced susceptibility to baloxavir marboxil were occasionally reported in patients [3,4,5]. The resistant virus has also caused influenza in humans [6,7].

Human H7N9 virus infections were first reported in March 2013 [8] and the virus was shown to possess amino acid mutations important for mammalian adaptation and to have the potential to transmit between ferrets via respiratory droplets [1,9,10,11]. During the fifth epidemic wave in the 2016–2017 influenza season, highly pathogenic H7N9 virus that retained the mammalian-adaptive mutations and the ability to transmit between ferrets was detected in both humans and chickens [2,12,13,14]. As of June 2019, a total of 1568 laboratory-confirmed human cases, including 615 deaths, have been reported [15]. Due to a nationwide domestic poultry vaccination campaign that began in September 2017 in China, human cases drastically decreased from mid-2017 [16]. However, the H7N9 viruses have not been eradicated because several outbreaks in poultry and a human infection by the highly pathogenic H7N9 virus were reported after the vaccination campaign [17].

## 2. Materials, Methods, and Results

To evaluate the efficacy of baloxavir marboxil in vivo infection with the H7N9 virus, baloxavir marboxil was orally administered to mice at 5 and 50 mg/kg twice a day for five days and was shown to have completely protected them from lethal challenge infection with a low pathogenic avian H7N9 human isolate, A/Anhui/1/2013 [18]. Highly pathogenic A/Guangdong/17SF003/2016 virus, which possesses enhanced polymerase activity in mammals due to PB2-482R, PB2-588V, and PA-497R [12] is more pathogenic than A/Anhui/1/2013 because it causes a systemic infection in mice, ferrets, and macaques [2]; this greater pathogenicity may affect the efficacy of baloxavir marboxil. Although A/Guangdong/17SF003/2016 showed reduced growth in human bronchial epithelial cells [2], this virus possesses the A100V, R262K, V387I, N394D, I465V, and K497R mutations in PA that may affect sensitivity to baloxavir marboxil compared with A/Anhui/1/2013. Accordingly, here, we assessed the efficacy of baloxavir marboxil against this highly pathogenic human H7N9 virus in vitro and in vivo.

**Figure 1:**
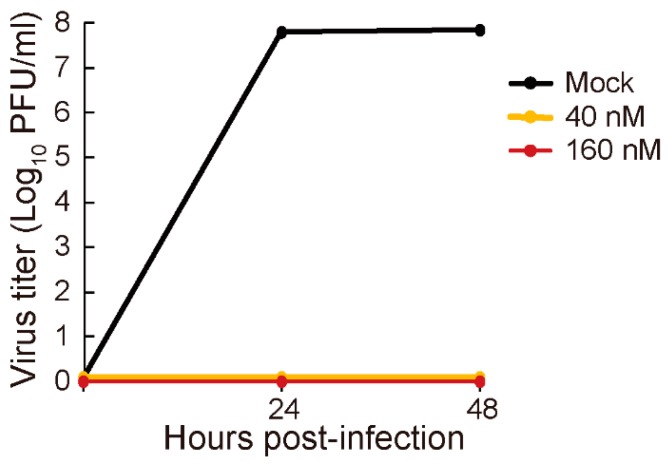
Virus growth kinetics in the presence of baloxavir marboxil. MDCK cells were infected with A/Guangdong/17SF003/2016 virus at an MOI of 0.001 and incubated with baloxavir marboxil at 0, 40, or 160 nM. At 24 and 48 h post infection, virus titers in the culture media were determined by means of plaque assays. Virus titers are expressed as means ± SD (*n* = 3).

To examine the efficacy of baloxavir marboxil in vitro, virus growth was tested in the absence or presence of baloxavir acid. Madin-Darby canine kidney (MDCK) cells were infected with A/Guangdong/17SF003/2016 (H7N9) virus at a multiplicity of infection (MOI) of 0.001 and incubated for 24 or 48 h with Eagle’s minimal essential medium (EMEM) supplemented with 0.3% BSA, 1 μg/mL trypsin, and 0, 40, or 160 nM baloxavir acid. Virus titers were measured by means of plaque assays with MDCK cells. A/Guangdong/17SF003/2016 grew efficiently in MDCK cells in the absence of baloxavir marboxil, reaching a virus titer of approximately 7.8 log_10_ PFU/mL at 24 and 48 h post infection (Figure 1). In contrast, in the presence of 40 and 160 nM baloxavir acid, A/Guangdong/17SF003/2016 replication was completely suppressed at both timepoints (Figure 1). These results indicate that baloxavir marboxil is effective against the highly pathogenic H7N9 virus in vitro.

**Figure 2:**
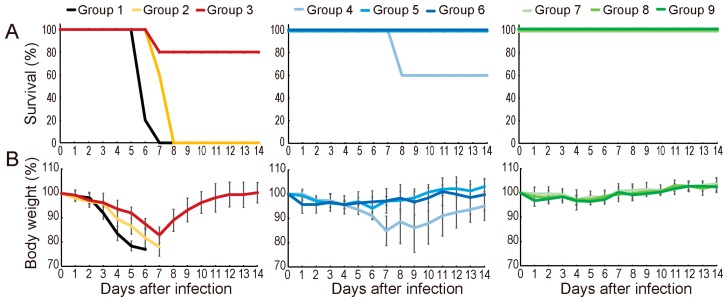
Treatment of infected mice with baloxavir marboxil. Mice were intranasally infected with 10 MLD_50_ of a highly pathogenic H7N9 virus. Five mice per group were treated as described in Table 1. Survival (**A**) and body weight changes (**B**) were monitored for 14 days post infection. Mouse body weights are expressed as mean ± SD.

Next, we assessed the efficacy of baloxavir marboxil in mice infected with the highly pathogenic human H7N9 virus. Six-week-old female mice (BALB/c, Japan SLC Inc.) were anesthetized with isoflurane and intranasally infected with 10 mouse lethal dose 50 (MLD_50_; 10^4.3^ PFU) of highly pathogenic A/Guangdong/17SF003/2016 (H7N9) possessing NA-294R (arginine at position 294 of NA indicates sensitive to NA inhibitors) [2,12]. Five infected mice per group were orally treated with oseltamivir phosphate at 5 or 50 mg/kg twice a day for 5 days or with baloxavir marboxil at 1.5, 15, or 50 mg/kg once or twice a day for 5 days (Table 1). The negative control mice received 0.5% methylcellulose because this reagent was used as a solvent. Body weight changes of these mice were monitored for 14 days and mice that lost 25% or more of their initial body weight were scored as dead and euthanized according to institutional guidelines. All animal experiments were conducted in accordance with the University of Tokyo’s Regulations for Animal Care and Use, which were approved by the Animal Experiment Committee of the Institute of Medical Science, the University of Tokyo (approval no. PA15-12). Mice given methylcellulose exhibited immediate body weight loss and died up to 8 days after infection (Figure 2A,B). Oseltamivir phosphate treatment at 5 mg/kg for 5 days slightly improved the survival time of the infected mice (*p* = 0.009, log-rank test) but failed to protect them from the lethal challenge infection (Figure 2A). Oseltamivir phosphate treatment at 50 mg/kg for 5 days showed 80% protection with severe body weight loss (Figure 2A,B). In contrast, 60% of mice treated once with baloxavir marboxil at 1.5 mg/kg survived for 14 days, whereas all of the mice in the other baloxavir-treated groups survived without any body weight loss (*p* = 0.0016, log-rank test). These results show that a single dose of baloxavir marboxil with 15 mg/kg is sufficient to protect mice from infection with a highly pathogenic human H7N9 virus.

To compare nasal turbinate and lung virus titers at three and six days post-infection of mice with 10 MLD_50_ of highly pathogenic A/Guangdong/17SF003/2016 virus during and after the various treatment regimens, we performed a standard plaque assay by use of MDCK cells. On day 3 post-infection, the virus titers in the nasal turbinates and lungs of mice treated with baloxavir marboxil were significantly decreased except for the virus titers in the lungs of mice treated with the single 1.5 mg/kg dose of baloxavir (Table 2). A similar trend was observed for the virus titers of the baloxavir marboxil-treated groups on day 6 post-infection. In particular, baloxavir treatment for five days strongly suppressed virus replication in the nasal turbinates and lungs. Treatment with oseltamivir phosphate at 5 or 50 mg/kg resulted in only a limited virus titer reduction in the nasal turbinates or lungs of mice on days 3 or 6 post infection (Table 2). These results show that baloxavir marboxil reduces the replication of highly pathogenic human H7N9 virus in the nasal turbinates and lungs. In addition, repeated treatments with baloxavir marboxil improve the therapeutic effect in the mouse model.

Emergence of drug-resistant mutants after antiviral treatment is one of the major concerns for antiviral treatment [19]. In fact, viruses resistant to baloxavir marboxil were reported in patients after baloxavir marboxil treatment [3,4,5]. To examine whether such mutants emerged in mice treated with baloxavir marboxil or oseltamivir phosphate, we determined the genome sequences of PA for the baloxavir marboxil-treated groups and of NA for the oseltamivir phosphate-treated groups by using a standard Sanger sequencing method. Briefly, viral RNA was extracted from lung samples of oseltamivir phosphate-treated mice or from the supernatant of cells infected with the virus that was plaque-purified from nasal turbinate or lung homogenates from baloxavir marboxil-treated mice. The rest of the lung homogenate samples that were used for virus titration were used for sequencing analysis. The cDNA was synthesized by using Superscript III reverse transcriptase (Life Technologies Japan Ltd., Tokyo, Japan) and a U12 primer (5′-AGCAAAAGCAGG-3′). The cDNA products were amplified by using Polymerase Chain Reaction (PCR) with Phusion High-Fidelity DNA Polymerase (New England Biolabs Japan Inc., Tokyo, Japan) and primers specific for the PA or NA segment. The PCR products were sequenced with a BigDye terminator 3.1 kit on an ABI 3130xl (Life Technologies Japan). We found no amino acid substitutions at position 38 of the PA of five plaque-purified viruses from nasal turbinate or lung samples from the baloxavir marboxil-treated mice at 6 days post infection and no amino acid substitutions in the NA of viruses isolated from the lungs of mice treated with oseltamivir phosphate at 3 and 6 days post infection (the samples analyzed were indicated by bolded numbers in Table 2).

## 3. Discussion

Here, we evaluated the in vivo therapeutic efficacy of baloxavir marboxil against a highly pathogenic human H7N9 virus. Baloxavir marboxil given as a single dose or as a 5-day treatment course improved the survival of mice infected with the highly pathogenic human H7N9 virus and suppressed its replication in the respiratory organs. Baloxavir marboxil also protected mice from a low pathogenic avian H7N9 human isolate, A/Anhui/1/2013 [18]. Since treatment with NA inhibitors have little antiviral effects against the low pathogenic and highly pathogenic human H7N9 viruses in mice [1,2], baloxavir marboxil would likely be the first choice to treat patients suffering from a H7N9 virus infection in the clinical setting.

Treating with antivirals always encounters the potential problem of emergence of resistant mutants. Viruses resistant to baloxavir marboxil frequently emerged in patients within 3–5 days of treatment [3,4,5] with the PA-I38T/M/F mutation known to increase the IC_50_ value by 10- to 50-fold [4,5]. In our experiments, viruses resistant to baloxavir marboxil or oseltamivir phosphate were not detected. However, to reduce the risk of emergence of resistant viruses, it is essential to minimize virus replication in the respiratory organs after treatment. To achieve this, treatment with high-dose baloxavir marboxil for several days would be a reasonable approach based on our data. This proposed treatment regimen is different from current baloxavir treatment for seasonal influenza; however, based on the relatively high frequency of viruses with reduced baloxavir sensitivity, even for seasonal influenza, a combination therapy of baloxavir marboxil and other antivirals for seasonal influenza may be considered as another therapeutic option to avoid the emergence of viruses with reduced baloxavir sensitivity.

In summary, baloxavir marboxil showed high therapeutic efficacy against a highly pathogenic human H7N9 virus. Since the risk of reemergence of H7N9 viruses persists, stockpiling baloxavir marboxil should be considered for pandemic preparation.

## Figures and Tables

**Table 1:** Summary of treatment groups.

Group No.	Compound	Concentration	Treatment Regimen ^a^
1	Methylcellulose	0.5%	–
2	Oseltamivir phosphate	5 mg/kg	Twice a day for 5 days
3	Oseltamivir phosphate	50 mg/kg	Twice a day for 5 days
4	Baloxavir marboxil	1.5 mg/kg	Once, 1 h post-virus inoculation
5	Baloxavir marboxil	15 mg/kg	Once, 1 h post-virus inoculation
6	Baloxavir marboxil	50 mg/kg	Once, 1 h post-virus inoculation
7	Baloxavir marboxil	1.5 mg/kg	Twice a day for 5 days
8	Baloxavir marboxil	15 mg/kg	Twice a day for 5 days
9	Baloxavir marboxil	50 mg/kg	Twice a day for 5 days

^a^ All treatments were administered orally.

**Table 2:** Lung virus titers of infected mice treated with the inhibitors.

Group No.	Virus titer (Mean Log_10_ PFU ± SD/g) ^a^			
	Day 3		Day 6	
	Nasal Turbinate	Lung	Nasal Turbinate	Lung
1	5.6 ± 0.3	7.2 ± 0.2	4.8 ± 0.4	5.3 ± 0.3
2	5.8 ± 0.2	**6.3 ± 0.1** **^c^**	4.4 ± 0.5	**4.9 ± 0.1**
3	5.5 ± 0.2	**6.5 ± 0.2**	4.2 ± 0.5	**4.8 ± 0.4**
4	3.9 ± 0.7 **	6.2 ± 0.2	**3.9**, <1.7, <1.7 *	**4.8 ± 0.7**
5	3.4, <1.7 ^b^, <1.7 **	3.5 ± 0.7 **	**5.1**, <1.7, <1.7	3.2 ± 0.5 **
6	2.6, <1.7, <1.7 **	3.1, 3.5, <1.7 **	2.8, <1.7, <1.7 *	**3.1**, 1.8, <1.7 **
7	3.1 ± 0.3 **	3.4 ± 0.7 **	**2.3**, <1.7, <1.7 **	**3.0**, <1.7, <1.7 **
8	2.5, 2.6, <1.7 **	<1.7, <1.7, <1.7 **	3.0, <1.7, <1.7 *	<1.7, <1.7, <1.7 **
9	2.6, <1.7, <1.7 **	<1.7, <1.7, <1.7 **	<1.7, <1.7, <1.7 **	<1.7, <1.7, <1.7 **

^a^ BALB/c mice were intranasally inoculated with 10 MLD_50_ of highly pathogenic human H7N9 virus (A/Guangdong/17SF003/2016). Three animals per group were euthanized on days 3 and 6 post infection. ^b^ Detection limit = 1.7 log_10_ PFU/g. ^c^ Bold numbers indicate that sequence analysis of NA or PA was performed. Statistically significant differences compared with the control group were determined by use of a one-way analysis of variance followed by a Dunnett test. * and ** indicate *p* < 0.05 and *p* < 0.01, respectively.
